# MMP8 increases tongue carcinoma cell–cell adhesion and diminishes migration via cleavage of anti-adhesive FXYD5

**DOI:** 10.1038/s41389-021-00334-x

**Published:** 2021-05-31

**Authors:** K. Juurikka, A. Dufour, K. Pehkonen, B. Mainoli, P. Campioni Rodrigues, N. Solis, T. Klein, P. Nyberg, C. M. Overall, T. Salo, P. Åström

**Affiliations:** 1 Cancer and Translational Medicine Research Unit, Faculty of Medicine, University of Oulu, Oulu, Finland; 2 Medical Research Center Oulu, Oulu University Hospital and University of Oulu, Oulu, Finland; 3 Department of Physiology & Pharmacology, University of Calgary, Calgary, Canada; 4 Department of Oral Biological and Medical Sciences, Faculty of Dentistry, Centre for Blood Research, and Department of Biochemistry and Molecular Biology, University of British Columbia, Vancouver, Canada; 5 Biobank Borealis of Northern Finland, Oulu University Hospital, Oulu, Finland; 6 Department of Oral and Maxillofacial Diseases, Faculty of Medicine, University of Helsinki, Helsinki, Finland; 7 Helsinki University Hospital, Helsinki, Finland; 8 Translational Immunology Research Program (TRIMM), University of Helsinki, Helsinki, Finland; 9 Research Unit of Biomedicine, Faculty of Medicine, University of Oulu, Oulu, Finland

**Keywords:** Cancer microenvironment, Cell adhesion, Extracellular matrix

## Abstract

Matrix metalloproteinases (MMPs) modify bioactive factors via selective processing or degradation resulting in tumour-promoting or tumour-suppressive effects, such as those by MMP8 in various cancers. We mapped the substrates of MMP8 to elucidate its previously shown tumour-protective role in oral tongue squamous cell carcinoma (OTSCC). MMP8 overexpressing (+) HSC-3 cells, previously demonstrated to have reduced migration and invasion, showed enhanced cell-cell adhesion. By analysing the secretomes of MMP8 + and control cells with terminal amine isotopic labelling of substrates (TAILS) coupled with liquid chromatography and tandem mass spectrometry (LC-MS/MS), we identified 36 potential substrates of MMP8, including FXYD domain-containing ion transport regulator 5 (FXYD5). An anti-adhesive glycoprotein FXYD5 has been previously shown to predict poor survival in OTSCC. Cleavage of FXYD5 by MMP8 was confirmed using recombinant proteins. Furthermore, we detected a loss of FXYD5 levels on cell membrane of MMP8 + cells, which was rescued by inhibition of the proteolytic activity of MMP8. Silencing (si) FXYD5 increased the cell-cell adhesion of control but not that of MMP8 + cells. siFXYD5 diminished the viability and motility of HSC-3 cells independent of MMP8 and similar effects were seen in another tongue cancer cell line, SCC-25. FXYD5 is a novel substrate of MMP8 and reducing FXYD5 levels either with siRNA or cleavage by MMP8 increases cell adhesion leading to reduced motility. FXYD5 being a known prognostic factor in OTSCC, our findings strengthen its potential as a therapeutic target.

## Introduction

Matrix metalloproteinases (MMPs) are a family of enzymes with a diverse substrate repertoire^1^ including structural macromolecules of extracellular matrix (ECM), cell surface receptors, growth factors, chemokines, and cytokines^2,3^. Their proteolytic activities modulate protein-protein interactions, determine fate and activity of proteins as well as participate in transducing cellular signals^4^. In cancer, MMPs promote tumourigenesis via breakdown of ECM, cleavage of cell adhesion proteins and impact cell signalling, thus enabling cancer growth and spread. Importantly, the proteolytic actions of MMPs on their targets may lead to alterations in protein functions and subsequently to diminished or accelerated cancer progression^5^.

MMP8, also known as collagenase-2 or neutrophil collagenase, was originally identified in neutrophils^6^ and possesses both tumour-suppressive and tumour-promoting effects depending on the cancer model or specific tissue where the cancer is located^7^. A number of MMP8’s substrates have been characterized but the molecular mechanisms as well as availability of substrates largely vary depending on tissues and diseases. Most of the molecular mechanisms of MMP8 were identified in breast^8^, prostate^9^, pancreatic^10^, gastric^11^, and liver^12^ cancers and include interactions with cell adhesion molecules and cleavage of cell signalling ligands and receptors.

Cell adhesion, including both cell-extracellular matrix and cell-cell adhesion, is crucial for the homoeostasis of healthy tissues. In cancer, the cell adhesion pattern shifts to favour cell motility – a key step in cancer cell invasion and metastasis^13^- and is mediated mainly via proteins from four major groups: cadherins, integrins, selectins and immunoglobulins^14^. In addition, other proteins such as FXYD domain-containing ion transport regulator 5 (FXYD5; dysadherin) belonging to the FXYD family of auxiliary subunits and regulators of Na-K-ATPase, participate in regulating cell adhesion via, for example, down-regulation of E-cadherin.

Oral tongue squamous cell carcinoma (OTSCC) is one of the most common oral malignancies^15–17^ but despite advances in treatment, the five-year survival is only around 65%^18^. One of the main reasons for the poor survival is high incidence of metastasis to neck lymph nodes, with greater frequency than in any other oral cancer type^19^. Importantly, metastases are estimated to cause up to 90% of all cancer specific deaths^20^. In human OTSCC, high MMP8 expression predicts better survival^21,22^, whereas high expression of FXYD5 worsens the patients’ prognosis^23^. Moreover, knock-out of *MMP8* in mice leads to increased susceptibility for carcinogen-induced tongue cancer^22^. We and others have shown that MMP8 decreases the migration and invasion of cancer cells in vitro^7^. Yet, the studies examining the role of FXYD5 in cancer cell behaviour are few.

In this study, we explored novel potential substrates of MMP8 to unravel the molecular basis for its tumour-protective effects in aggressive OTSCC. Moreover, we examined the functional role of FXYD5, identified as MMP8 substrate, on OTSCC cell behaviour.

## Results

### MMP8 increases the cell-cell adhesion

**Fig. 1:**
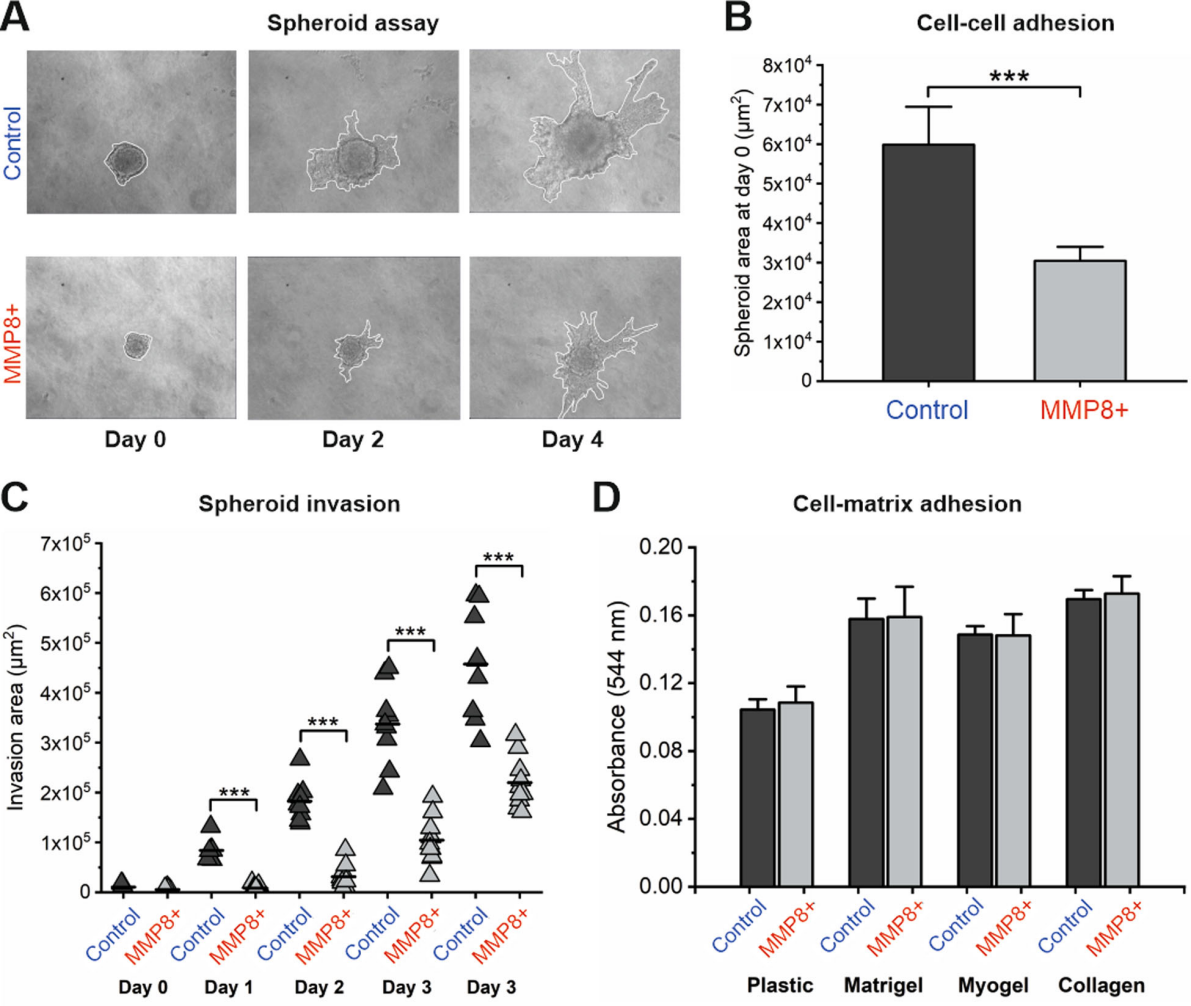
The cell–cell adhesion, cell–matrix adhesion and invasion of control and MMP8 + HSC-3 cells. **A** Representative images of spheroids in Myogel–Fibrin matrix. White line denotes the whole area of the spheroids. **B** The spheroid area of control and MMP8 + cells embedded in Myogel–Fibrin matrix at day 0 (number of spheroids analysed = 9–10, experiment was repeated three times). ***p ≤ 0.001. **C** The invasion area changes over the duration of 4 days for control and MMP8 + cells embedded in Myogel–Fibrin matrix (number of spheroids analysed = 9–10, experiment was repeated three times). ***p ≤ 0.001. **D** The cell–matrix adhesion of control and MMP8 + cells to plastic, Matrigel, Myogel and collagen type I. Data is presented as average of four individual experiments.

Cell-cell and cell-matrix adhesion facilitates cell migration and as MMP8 inhibits the migration of various cancer cells^21^, we wanted to investigate if MMP8 regulates the adhesion of OTSCC cells. The area of newly formed spheroid was (representative images shown in Fig. 1A) used as an indicator of cell-cell adhesion^24^ (at later time points proliferation also affects spheroid size). The area of spheroid formed by MMP8 overexpressing (MMP8 + ) HSC-3 cells were significantly smaller in size (Fig. 1B) than those of control cells suggesting that MMP8 enhances cell–cell adhesion of OTSCC cells. The spheroids kept the smaller size in all time points examined (data not shown, representative images in Fig. 1A). Furthermore, the invasive area was significantly smaller in spheroids of MMP8 + HSC-3 cells in all timepoints examined after the time point 0 h (Fig. 1C), strengthening our previous findings of the anti-invasive effects of MMP8 in OTSCC^21,22^. Importantly, cell-matrix adhesion to plastic, Matrigel, Myogel^25^ or collagen I was unaffected in MMP8 + HSC-3 cells as compared to control cells (Fig. 1D). Thus, MMP8 regulates OTSCC cell-cell interactions but not cell-matrix interactions.

### N-terminomics/TAILS analysis revealed potential novel substrates of MMP8

**Fig. 2:**
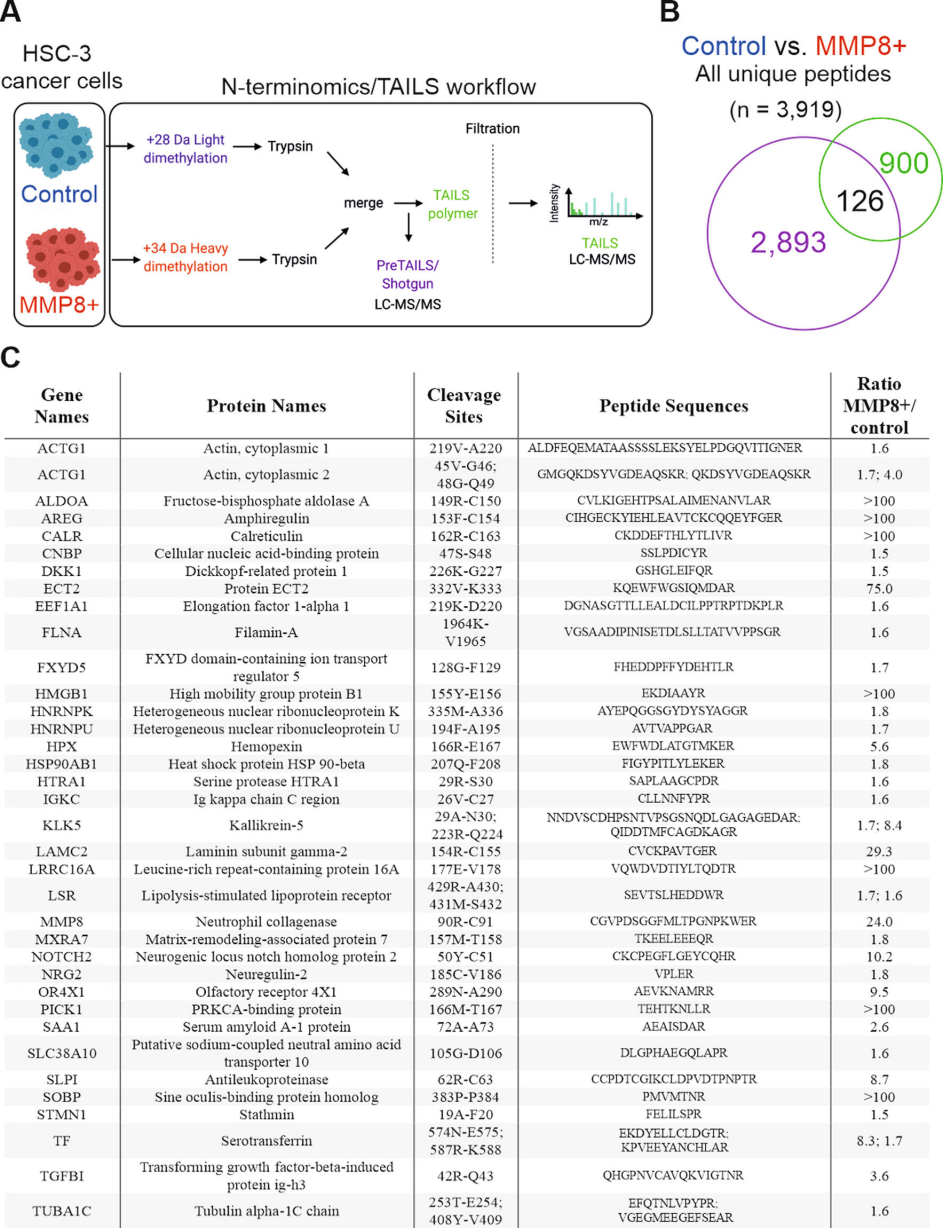
Peptides identified by pre-enrichment TAILS and TAILS experiments in MMP8 + versus control HSC-3 cells. **A** Schematic of the N-terminomics/TAILS workflow. MaxQuant was used at 1% false discovery rate (FDR) to analyse the data. **B** Venn diagram depicting unique peptides identified in the pre-enrichment TAILS and TAILS samples. **C** Peptides elevated in the MMP8 + conditioned media and analysed with MaxQuant and TopFINDER. Candidate MMP8 substrates are shown with their potential cleavage sites.

To characterize the substrates in cell secretomes potentially cleaved by MMP8 (overexpressed in HSC3 cells), we utilized terminal amine isotopic labeling of substrate (TAILS)^26–29^, a N-terminomics technique that enriches for natural N-termini and neo-N-termini. Additionally, we performed shotgun proteomics analysis (Fig. 2A). 3,919 unique peptides were identified in the conditioned media: 900 unique peptides were identified by the TAILS analysis, 2,893 unique peptides were identified in the pre-enrichment TAILS samples, and 126 peptides were identified in both analysis (Fig. 2B). This data demonstrated that TAILS analysis successfully enriched for unique N-termini peptides. Analysis of the amino acid composition in the proximity of the cleavage sites revealed leucine and lysine as most common amino acids residing in the substrates of MMP8 in P1 position, whereas serine was the most common amino acid in P1 position of proteins cleaved by proteases in control cells (Figure S1A). Importantly, MMP8 + cells have a comparable preference profile of MMP8 cleavage specificity for proline at P3, leucine at P1′, leucine at P2′, glycine at P3′ as we demonstrated previously^30^. The preference for lysine at P1 could be due to other proteases present in the conditioned media of MMP8 + cells such as kallikrein-5 (KLK5) (Fig. 2C). Of the 900 unique peptides identified by TAILS, a total of 36 potential MMP8 substrates were identified (Fig. 2C). Using TopFIND and PathFINDer^26,31,32^, the MMP8 protease web was generated (Figure S1B) demonstrating potential cleavage networks. Importantly, MMP8 is known to cleave KLK5^33^ at position ^223^R↓Q^224^ based on TopFIND and PathFINDer, therefore suggesting a synergistic and downstream regulation by MMP8 via KLK5.

### Pre-enrichment TAILS analysis revealed differentially expressed proteins in control and MMP8+cells

**Fig. 3:**
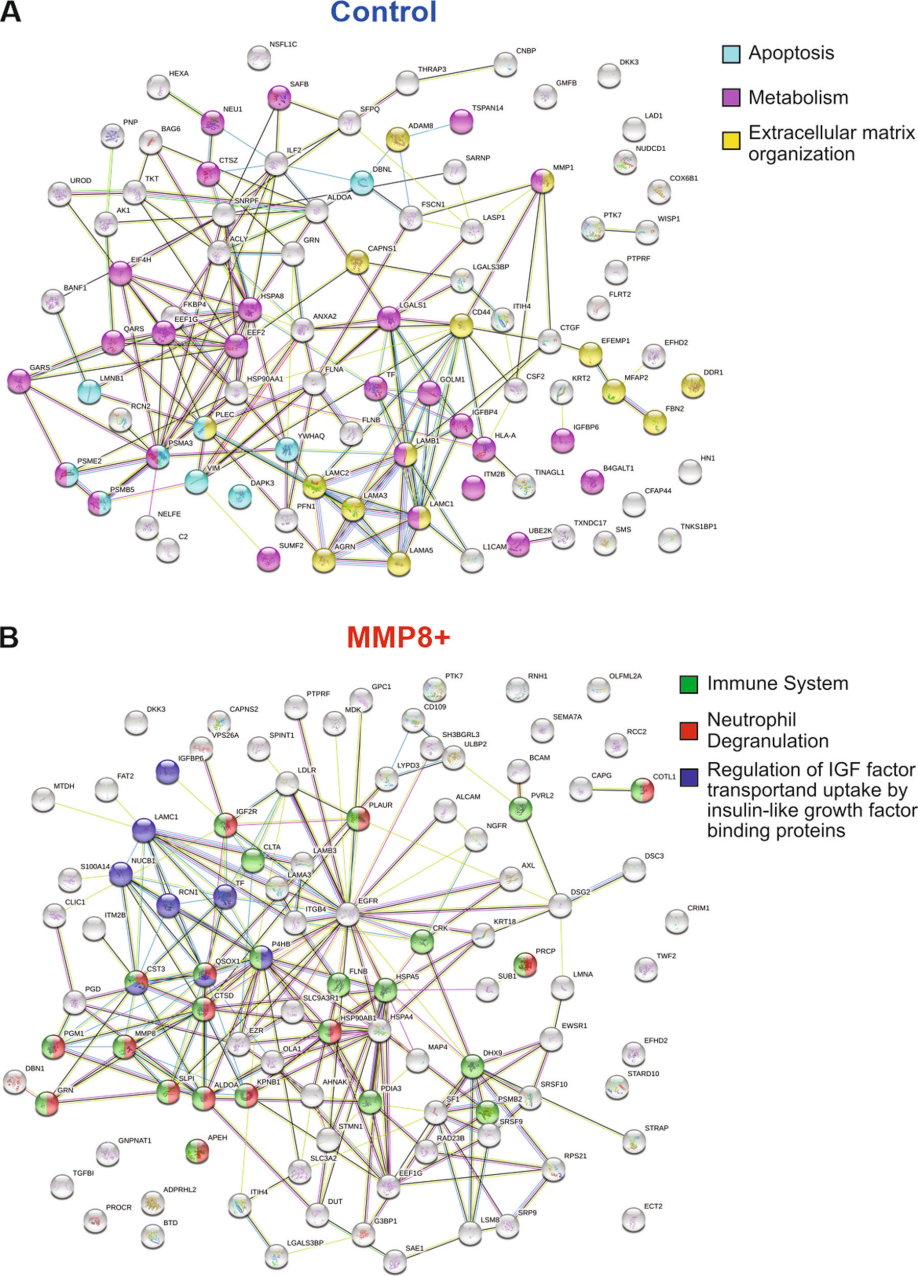
Analysis of protein−protein interaction networks. Significantly elevated proteins from (**A**) control or (**B**) MMP8 + HSC-3 cells conditioned media identified from the pre-enrichment TAILS analysis were compared using STRING-db v11 software^68^ using a 5% false discovery rate. Coloured lines between the proteins indicate different types of interaction evidence: known interactions (teal), experimentally determined (pink), predicted interactions gene neighbourhood (green), gene fusions (red), gene co-occurrence (blue), text-mining (yellow), coexpression (black), protein homology (purple).

All the peptides identified in the secretomes of MMP8 + and control HSC-3 analysed with LC–MS/MS are presented in Supplementary table S1 and those analysed with TAILS in Supplementary table S2. Interestingly, MMP8 appears to undergo autocatalytic cleavage as the peptide ^91^CGVPDSGGFMLTPGNPKWER^110^ was increased in the MMP8 + cells as compared to control. In the shotgun/pre-enrichment TAILS samples, 3,019 unique peptides (corresponding to 1,681 proteins) were identified in the HSC-3 cell secretomes and the significantly changing proteins were subjected to STRING-db analysis. In the control cells, an enrichment for apoptosis, metabolism and extracellular matrix organization was identified using STRING-db (Fig. 3A). In contrast, an enrichment for immune system, neutrophil degranulation and regulation of IGF factor transport and uptake by insulin-like growth factor binding proteins was found in MMP8 + cells (Fig. 3B). To further characterize the substrate repertoire, we performed TopFINDER analysis. Substrates identified in control and MMP8 + HSC-3 cells, including peptides, cleavage sites, and P10 to P1 and P1′ to P10′ positions, are shown in Supplementary tables S3 and 4.

**Fig. 4:**
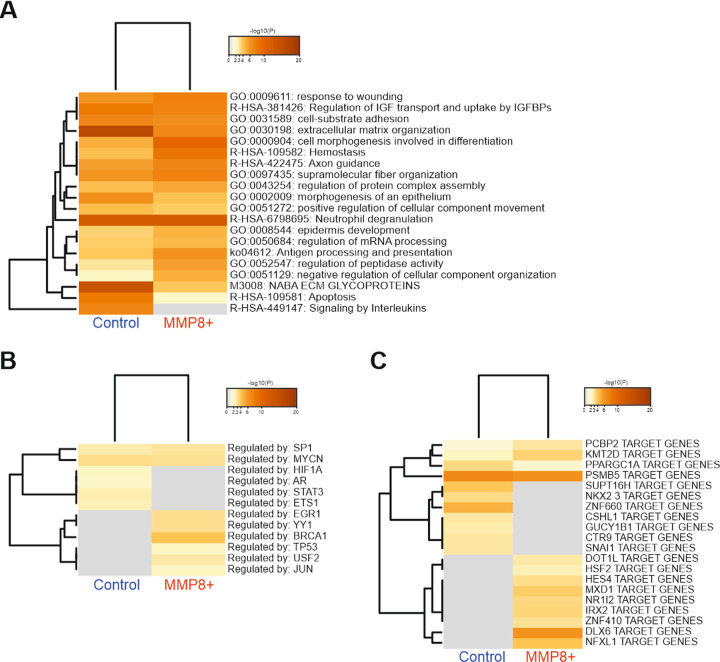
Changes in protein expression in control and MMP8 + HSC-3 cells’ secretomes from pre-enrichment TAILS data. **A** Heatmap, differentially expressed proteins in control and MMP8 + HSC-3 cells as analysed using Metascape. **B** Heatmap, comparison of genes known to regulate the significantly elevated proteins in control and MMP8 + HSC-3 cells as analysed using Metascape. **C** Heatmap, the comparison of regulated pathways known to regulate the differentially expressed genes in control and MMP8 + HSC-3 cells as analysed using Metascape.

Using Metascape analysis (https://metascape.org), multiple comparisons and enrichment of Gene Ontology (GO)-annotated protein groups (Fig. 4A), transcriptional factors (Fig. 4B) and target genes (Fig. 4C) in control and MMP8 + cells are depicted in heatmaps. Comparison of GO-annotated protein groups showed that the conditioned media of control cells expressed elevated proteins linked to changes in extracellular matrix organisation, apoptosis and interleukin signalling as compared to MMP8 + conditioned media. Interestingly, the number of apoptotic cells was higher in MMP8 + HSC-3 cells compared to controls (35.6% vs. 19.1% respectively, Fig. S2). In MMP8 + cells, an enrichment for regulation of peptidase activity was identified. Thus, the expression of MMP8 regulates multiple biological functions in HSC-3 cells.

### MMP8 cleaves FXYD5

**Fig. 5:**
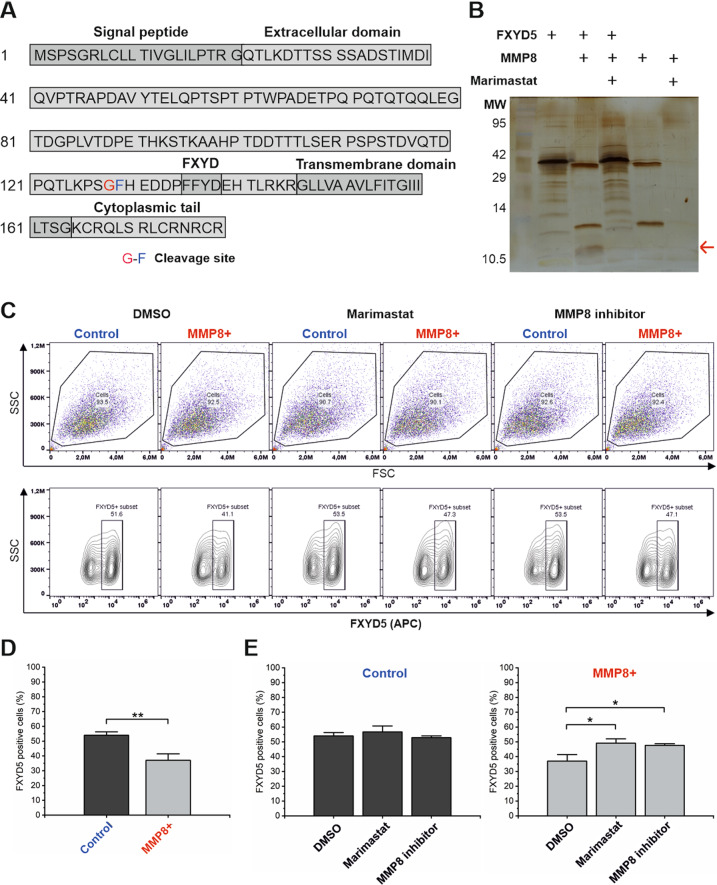
FXYD5 is a substrate of MMP8. **A** Cleaved FXYD5 peptide identified by the TAILS experiment. Modified from UniProt query Q96DB9. **B** Cleavage assay of recombinant FXYD5 with and without APMA-activated recombinant MMP8 using silver staining. 10 µM Marimastat was used to inhibit MMP8. **C** Expression of FXYD5 on the membrane of MMP8 + and control HSC-3 cells after treatment with 10 µM Marimastat, 10 µM MMP8 specific inhibitor or DMSO (vehicle control) as analysed by flow cytometry. Representative graphs are shown. **D** Comparison of membrane FXYD5 expression in control and MMP8 + HSC-3 cells as analysed by flow cytometry. Data are presented as the average of three individual experiments. **p ≤ 0.01. **E** Comparison of membrane FXYD5 expression with 10 µM Marimastat or MMP8 specific inhibitor as analysed by flow cytometry. Data are presented as the average of three individual experiments. *p ≤ 0.05.

Because MMP8 affected cell–cell adhesion, from novel potential substrates, we focused to validate the cleavage of anti-adhesive membrane glycoprotein FXYD5, also known as dysadherin, by MMP8. The identified cleavage site of FXYD5 (^128^G↓F^129^) by MMP8 identified by TAILS is located in the extracellular domain of FXYD5 close to the transmembrane domain as illustrated in Fig. 5A. The cleavage was verified by an in vitro cleavage assay (Fig. 5B), which demonstrated that APMA-activated recombinant MMP8 mostly degraded recombinant FXYD5 leaving only peptides smaller than 10 kDa present and the cleavage was inhibited by broad-spectrum MMP inhibitor Marimastat. Some MMPs^34^, including MMP8, undergo autolysis which is inhibited by Marimastat as seen in Fig. 5B. MMP8 cleaved FXYD5 at enzyme:substrate (E:S) ratios of 1:10–1:100, but the cleavage was not detectable at E:S 1:500 (Fig. S3).

TAILS analysis detected elevated levels of FXYD5 fragments in secretomes of MMP8 + cells, suggesting that it is cleaved from the membrane of MMP8 + cells. Accordingly, flow cytometry analysis (Fig. 5C) showed that MMP8 + HSC-3 cells display approximately 30% less FXYD5 protein on the cell membrane compared to control cells (Fig. 5D). The addition of 10 µM of Marimastat, or MMP8 specific inhibitor recovered the levels of FXYD5 in MMP8 + cells close to the level of control cells (Fig. 5E). Tumoural FXYD5 expression is reported to inversely correlate with E-cadherin expression in many cancers^23,35–39^, however we found only minimal decrease in the level of E-cadherin in MMP8 + HSC3 cells compared to control cells. The membrane interactor of E-cadherin, β-catenin, showed slightly elevated levels in MMP8 + HSC-3 cells compared to control cells (Figure S4A-B). E-cadherin recruits β-catenin to the cell membrane and they form complexes to create adherens junctions crucial for the cell-cell adhesion. The E-cadherin and β-catenin seemingly co-localised more on the cell membranes of MMP8 + HSC-3 cells compared to control cells, as illustrated by the orange co-localisation signal in the Figure S4C.

### Silencing FXYD5 increases the cell–cell adhesion of control but not MMP8+cells

**Fig. 6:**
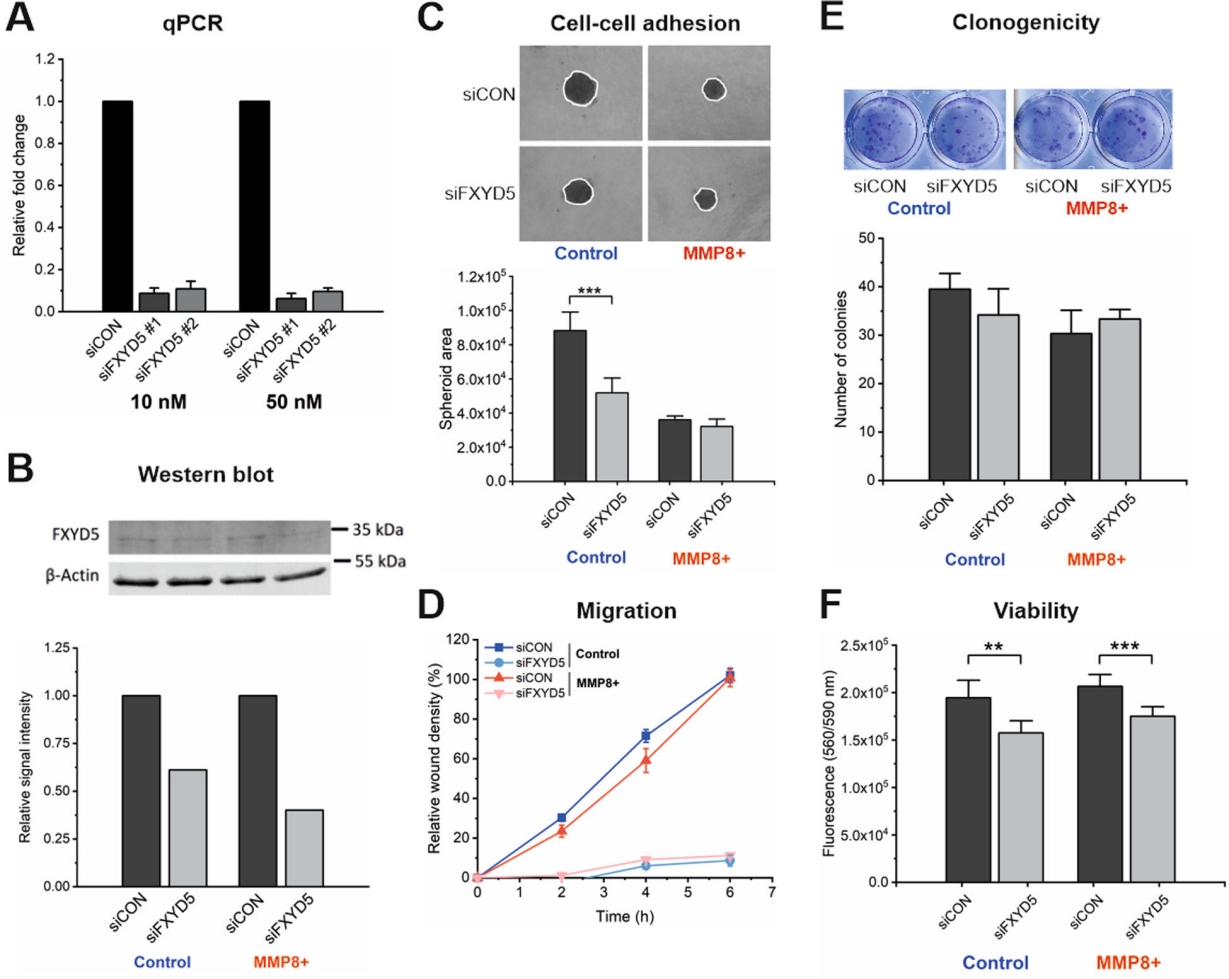
The effects of silencing FXYD5 were studied in control and MMP8 + HSC-3 cells. **A** FXYD5 gene expression analysed by qPCR in 10 nM and 50 nM siCON and siFXYD5 HSC-3 control cells. **B** FXYD5 protein expression analysed by western blot in whole cell lysates of siCON and siFXYD5 control and MMP8 + HSC-3 cells. **C** Cell–cell attachment evaluated as spheroid area in siCON and siFXYD5 control and MMP8 + HSC-3 cells (number of spheroids analysed = 4–6, experiment was repeated three times). White line denotes the whole area of the spheroids. ***p ≤ 0.001. **D** Migration as analysed by IncuCyte scratch wound assay of double-silenced siCON and siFXYD5 control and MMP8 + HSC-3 cells (number of replicates analysed = 4–6, experiment was repeated three times). **E** Colony formation as evaluated by clonogenic assay of siCON and siFXYD5 control and MMP8 + HSC-3 cells (number of replicates analysed = 6, experiment was repeated three times). Above are representative images. **F** Viability of siCON and siFXYD5 control and MMP8 + HSC-3 cells as analysed with resazurin assay (number of replicates analysed = 6, experiment was repeated three times). **p ≤ 0.01, ***p ≤ 0.001.

FXYD5 is an anti-adhesive factor and its levels were lower in MMP8 + cells with increased adhesion. Hence, we expected that silencing FXYD5 in control cells would increase their cell–cell adhesion. There were no significant differences in FXYD5 mRNA levels between MMP8 + and control cell line (data not shown) suggesting that the observed decrease in protein level is not due differential expression of FXYD5 in MMP8 + cells. RNA interference reduced the FXYD5 mRNA levels ~90% with both siRNAs and concentrations (Fig. 6A). On the protein level the reduction of FXYD5 was around 40% in control and 60% in MMP8 + HSC-3 cells (Fig. 6B). In whole cell lysates, detecting the total FXYD5 (membrane and cytosolic), MMP8 + cells did not show significantly lower levels of FXYD5 compared to the control cells (Fig. 6B), although flow cytometry analyses consistently indicated reduced levels on cell membrane (Fig. 5C).

As we expected based on the known function of FXYD5, silencing increased cell–cell adhesion in control cells leading to a spheroid with similar size to MMP8 + cells (Fig. 6C). Instead, further diminishing of spheroid size was minimal in MMP8 + HSC-3 cells after silencing FXYD5, suggesting that MMP8 is responsible for diminishing FXYD5 levels and abrogate its anti-adhesive function.

### Silencing FXYD5 halts the migration of OTSCC cells

Silencing of FXYD5 dramatically diminished the cell movement of both control and MMP8 + cell lines (Fig. 6D) beyond the anti-migratory effect of MMP8 illustrating the importance of cell–cell adhesion as well as FXYD5 expression for OTSCC motility. We did not find a significant effect on proliferation^21^ (data not shown) or clonogenicity on HSC-3 cells due to FXYD5 silencing or MMP8 overexpression (Fig. 6E). The viability, however, was diminished after FXYD5 silencing in both cell lines (Fig. 6F). The effects of FXYD5 silencing on OTSCC function was confirmed and re-producible in another aggressive tongue carcinoma cell line, SCC-25, which showed dramatic decrease in migration but also a significant effect on proliferation and clonogenicity (Fig. S5).

## Discussion

Previous studies report tumour-suppressive effects of MMP8 in breast, skin and tongue cancers and include various mechanisms affecting tumourigenesis, migration, invasion and metastasis^7^. Here, we used the N-terminomics/TAILS to characterize and identify novel MMP8 substrates in tongue cancer cells. Our study expands the knowledge on the function of MMP8, a protease expressed by various cell types and playing a role in a variety of physiological and pathological conditions^4,40^.

The role of MMP8 in cancer has been demonstrated in knock-out mice which were more susceptible for developing cancers of skin^41^, particularly melanoma^42^, breast^43^, and tongue^22^. Mouse models suggest that high MMP8 expression reduced metastasis formation in melanoma and breast cancer^8,42,44,45^ and diminished the migratory and invasive potential of melanoma and breast cancer cells in vitro. In tongue cancer, we have previously shown similar effects of MMP8 on carcinoma cell behaviour in vitro and in vivo^21,22^. One key element for cancer invasion and metastasis, and a prerequisite for malignant progression^14^, is the loss of cell adhesion to adjacent cells or to matrix^13^. Enhanced cell-matrix adhesion to fibronectin, collagen type I and laminin-1 was reported in MMP8 overexpressing human breast myoepithelial^46^ and murine melanoma cell lines^42^. Here we found that in tongue cancer cells, cell-matrix adhesion was unaffected by MMP8, but instead, enhanced cell-to-cell adhesion was observed, indicating molecular mechanisms different from that reported for the other cancers. The likely explanation for the stronger adhesion and subsequent diminished migration is our finding that MMP8 cleaves FXYD5, an anti-adhesive glycoprotein, from the cell membrane. FXYD protein family members have never been reported to be cleaved or degraded by any MMPs before this study. Our finding supports a novel tumour-protective molecular mechanism of MMP8 in OTSCC, which could apply to other diseases as well. However, previously reported substrate cleavage by MMP8 leads to various effects depending on the cancer type and it is possible that the mechanisms are disease specific. In breast cancer cells, MMP8 was shown to cleave decorin resulting in a miRNA-mediated signalling cascade and subsequent increase in anti-tumourigenic factors such as programmed cell death protein 4 (PDCD4)^8^. MMP8 also inactivates integrin beta-1 resulting in reduced invasiveness in multiple cancer cell lines, with the most prominent effect seen in prostate cancer^9^. However, in liver cancer cells MMP8 activates PI3K/Akt/Rac1 signalling pathway leading to increased aggressiveness^12^. In pancreatic and gastric cancer, Ephrin-B1 induces the secretion of MMP8 and is later cleaved by MMP8 as a feedback loop. Ephrin-B1 is a transmembrane ligand of Eph receptors crucial for cell migration, adhesion and cell-cell signalling and cleavage by MMP8 was shown to contribute to the aggressiveness, e.g., invasion potential, of the cancer cells^10,11^. Furthermore, other proteases may work together with MMP8 to facilitate its effects in various cancers as demonstrated in the protease web generated by PathFINDer (Fig. S1B). In this paper we show that the expression levels of proteases such as cathepsin D, cathepsin L, kallikrein-5 or serine protease HTRA1 are elevated in the secretomes of MMP8 + OTSCC cells. The detection of arginine in the P1 position in cleavage sites is likely due to enhanced expression of cathepsin L or kallikrein-5, which are known to prefer arginine in this position.

In cancers of the head and neck region, high expression of FXYD5 predicts poor prognosis in oesophageal squamous cell carcinoma^47^ and correlates with metastasis in head and neck squamous cell carcinoma^38^. Accordingly, a study by Nakanishi et al.^23^ showed that high FXYD5 protein expression in OTSCC tumours correlates with infiltrative growth pattern, high TNM stage and poor survival. Here we examined, for the first time, the functional effects of FXYD5 in OTSCC cells and found that diminishing the level of FXYD5 either with RNA interference or proteolytic action of MMP8, the migration of OTSCC cells reduces. Our attempts to stain FXYD5 in OTSCC patient tumour samples (described in the Supplementary methods) unfortunately technically failed as the staining was non-specific and as a result the data obtained was unusable for meaningful interpretation (data not shown). The antibody used by Nakanishi et al. showed membrane-localized staining but unfortunately was not available commercially, hence we could not reproduce the tumour sample analysis in our patient samples. High tumoural FXYD5 expression on the cell membrane has been shown to predict poor prognosis also in breast cancer^36^, epithelioid^48^ and synovial^49^ sarcomas, non-small cell lung cancer^50,51^, cervical cancer^52^ and serous ovarian carcinoma^53^, and in addition to correlate with metastasis in ductal pancreatic adenocarcinoma^35^, gastric cancer^54^, colorectal cancer^55^, cutaneous malignant melanoma^56^, thyroid carcinoma^37^ and extrahepatic cholangiocarcinoma^57^. FXYD5 expression has been previously connected to regulation of cell motility, metastasis and stem cell- likeproperties. Hence, our results in OTSCC cells strengthens the previous findings for the tumour-promoting role of FXYD5 in other cancers. Breast cancer cells overexpressing FXYD5 metastasize more compared to controls^36^ and similar findings were shown in ovarian cancer^58^. FXYD5 also disperses the TβR1 complex leading to enhanced TGF-β signalling and SMAD3/4 activation, which in turn upregulates FXYD5 gene expression in ovarian cancer^58^. In line, TGF-β treatment induced FXYD5 expression in endometrial cancer, which in turn led to activation of NF-κβ pathway^59^. Furthermore, in breast cancer cell lines, high FXYD5 expression correlates with activation of AKT signalling, which drives the EMT^60^. In hepatocellular carcinoma FXYD5 expression was linked to stem cell like properties, decreased apoptosis and higher tumour initiation in mice^61,62^. Here, we detected higher number of cells in early and late apoptosis in MMP8 + HSC-3 cells compared to controls. Also, apoptosis related proteins were upregulated in cleaved fragments found in control HSC3 cells compared to MMP8 + cells, suggesting changes in regulation of apoptosis. However, in none of our experiments we were able to detect any differences in cell proliferation^21^ or viability due to MMP8 overexpression.

Overall, our study indicates that FXYD5 is a novel substrate of MMP8 and its cleavage from cell membrane by MMP8 represents a previously unknown mechanism on how MMP8 increases cell-cell adhesion leading to restrained migration. Importantly, reducing the membrane FXYD5 expression as such, the tongue cancer cell-cell adhesion increases with subsequent remarkable reduction in mobility. Additional experiments are needed to further characterize the function of FXYD5 in vivo and the cleavage by neutrophil or tumour-derived MMP8 should be considered in experimental settings. As FXYD5 serves as a marker of worse prognosis in multitude of cancers, including OTSCC, our results suggest it as a promising therapeutic target also for tongue cancer. Furthermore, a therapeutic antibody against FXYD5 (M53, Creative Biolabs®) is available for research and should be considered for further studies.

## Materials and methods

### Cell culture and RNA interference

The creation of MMP8 overexpression (MMP8 + ) cell lines and culture conditions for human OTSCC cell lines HSC-3 (Japan Health Sciences Foundation, Tokyo, Japan) and SCC-25 (American Type Culture Collection, Manassas, VA, USA) have been described previously^21^. Two Ambion™ Silencer™ Select validated siRNAs against FXYD5 (ID s28765, “FXYD5 siRNA #1” and ID s28764, “FXYD5 siRNA #2”, siFXYD5) and negative control #1 siRNA (siCON, all from Thermo Scientific, Carlsbad, CA, USA) were used for RNA interference. The silencing was performed in 24-well (100 000 cells per well) or 6-well plates (250 000 cells per well) depending on the downstream experiment. The next day, the siRNAs were added to the plate at two different concentrations, 10 nM and 50 nM along with Lipofectamine RNAiMAX (Invitrogen, Carlsbad, CA, USA) transfection reagent following manufacturer’s instructions. After 48 h, RNA was extracted, and qPCR analyses were performed as described below. Based on the results of qPCR analysis, FXYD5 siRNA #1 at 10 nM concentration was applied for functional assays. Where indicated, the siRNA treatment was performed as described above for the second time after two days from the first silencing.

### Collection of cell secretomes and lysates

The cells grown to 80% confluency in T175 flasks were washed with PBS and cultured in Opti-MEM® without phenol red (Life Technologies, Paisley, UK) for 24 h. The conditioned media were collected, protease inhibitors (cOmplete™ Protease Inhibitor Cocktail tablets, Roche Diagnostics, Mannheim, Germany) added, and media clarified by centrifugation (1500 × *g* for 10 min) following filtering through a 0.22 µm filter. The buffer was exchanged to 50 mM HEPES, 150 mM NaCl and 10 mM CaCl_2_ including protease inhibitor as before using a 3 kDa cut-off membranes (Amicon® Ultra, Millipore, Carrigtwohill, Ireland). The amount of total protein was measured with DC protein assay (Bio-Rad Laboratories, Hercules, CA, USA) per manufacturer’s instructions and the samples were lyophilised.

The whole cell lysates were collected from 6-well plates for Western blot analyses. After washing twice with cold PBS, the proteins were extracted in RIPA buffer (25 mM Tris-HCl pH 7.6, 150 mM NaCl, 1% Triton X-100, 1% sodium deoxycholate, 0.1% SDS), supplemented with protease (cOmplete™ Protease Inhibitor Cocktail tablets, Roche Diagnostics) and phosphatase (PhosSTOP™, Sigma-Aldrich, St. Louis, MO, USA) inhibitors and incubated a minimum of 3 h shaking at 4 °C. Cell debris was removed by centrifugation (17,000 × *g* for 10 min at 4 °C) and protein concentration was measured with DC protein assay (Bio-Rad Laboratories) per manufacturer’s instructions.

### N-terminomics/TAILS and proteomics analyses

Lyophilised secretome samples were resuspended in 3 M guanidine HCl (pH 8.0) and 500 mM HEPES (pH 8.0). Protein denaturation was achieved with addition of 5 mM DTT and incubation at 37 °C for 60 min. Alkylation was done by adding 15 mM iodoacetamide and incubation in the dark at room temperature for 30 min and quenched with 15 mM DTT. Sample pH was adjusted to 6.0 with HCl. Next, α- and ε-amines were labelled with either 40 mM isotopically heavy ^13^CD_2_O or light CH_2_O labels by incubation with 1 M NaBH_3_CN overnight at 37 °C. Next the samples were combined and were precipitated using acetone/methanol (8:1). The resulting pellet was resuspended in 1 M NaOH and the proteins were subjected for trypsination with overnight incubation at 37 °C. For pre-enrichment TAILS, 10% of the sample was taken, and the pH adjusted between 2.0 and 3.0 with 100% acetic acid. The rest of the samples underwent the enrichment of N-termini for TAILS analysis as described previously^28,29^. Briefly, the samples were incubated overnight at 37 °C with a 3-fold excess (w/w) of dendritic polyglycerol aldehyde polymer with pH adjusted between 6.0 and 7.0 with 1 M HCl. To separate unbound peptides from polymer-bound peptides the samples were filtered with centrifugal filter units with 10-kDa cut-off membranes (Amicon® Ultra, Millipore). The flow-through was collected and the membrane washed with 100 mM Tris. The pH of the samples was adjusted between 2.0 and 3.0 with 100% acetic acid and the samples were stored on C18 stage tips until analysis.

The liquid chromatography and tandem mass spectrometry analysis were performed on an Impact II ultra-high-resolution quadrupole time-of-flight mass spectrometer (Bruker, Billerica, MA, USA), interfaced with an EASY-nanoLC 1000 (Thermo Fisher Scientific) using a CaptiveSpray nanoBooster ionization interface and a 75 µm × 400 mm analytical column of C18 1.8 µm resin with the column temperature at 50 °C as described earlier^63^. Peptide sequences were identified from the human UniProtKB/Swiss-Prot database containing 42,197 canonical and isoform protein sequences (downloaded August 2017) with appended standard laboratory and common contamination protein entries and reverse decoy sequences using the Andromeda algorithm as implemented in the MaxQuant software package v1.6.0.1, using a peptide false discovery rate (FDR) of 1%. Search parameters included a mass tolerance of 1 ppm for the parent ion and 0.5 Da for the fragment ions, carbamidomethylation of cysteine residues (+57.021464 Da), variable N-terminal modification by acetylation (+42.010565). N-terminal and lysine heavy (+34.063116 Da) and light (+28.031300 Da) dimethylation was defined as labels for relative quantification. The cleavage site specificity was set to semi-ArgC (search for free N-terminus) for the TAILS data and was set to ArgC for the preTAILS data, with up to two missed cleavages allowed. Significant outlier cutoff values were determined after log(2) transformation by boxplot-and-whiskers analysis using the BoxPlotR tool^64^. Database searches were limited to a maximal length of 35 residues per peptide. Peptide sequences matching reverse or contaminant entries were removed.

### Reactome Pathway Analysis

To identify interconnectivity among proteins, the STRING-db (Search Tool for the Retrieval of Interacting Genes) database was used. The protein-protein interactions are encoded into networks in the STRING v11 database (https://string-db.org). Metascape (https://metascape.org) analysis was used to identify changes in functional enrichment, interactome analysis, and gene annotation^65^. Our data were analysed using *Homo sapiens* as our model organism at a false discovery rate of 5%.

### Heatmaps of cleavage sites, TopFIND and PathFINDer analysis

WebPICS was used from the website http://clipserve.clip.ubc.ca/pics. TopFIND and PathFINDer analyses were performed using the website http://clipserve.clip.ubc.ca/topfind/. Bioinformatics searches were performed as described previously^26^.

### Cleavage assay

Recombinant MMP8 protein (R&D Systems, Minneapolis, MN, USA) was activated for 15 min with 1 mM 4-aminophenylmercuric acetate (APMA). After activation, broad spectrum MMP inhibitor Marimastat (Sigma-Aldrich) was added at 10 µM concentration for 1 h. Lastly recombinant human FXYD5 (Abcam, Cambridge, UK) was added with molar enzyme:substrate (E:S) ratios of 1:10–1:500 (starting from MMP8 concentration of ~400 nM) and incubated overnight at 37 °C. The reactions were stopped by adding 4x sample buffer (final concentrations 2 M urea, 0.5% SDS, 0.125 M Tris-HCl, bromophenol blue) and β-MeOH and the samples were boiled for 5 min. Cleavage products were separated on a 15% tris-glycine or 16% tris-tricine gel and visualised by silver staining.

### RNA extraction and qPCR

RNA was extracted with TRIzol™ Plus RNA Purification kit (Ambion™, Thermo Scientific) according to the manufacturer’s instructions. RNA quantity and quality were measured with NanoDrop 2000 (Bio-Rad Laboratories). cDNA was generated from up to 1 µg of RNA with RevertAid First Strand cDNA synthesis kit (Thermo Scientific). qPCR analysis was performed with FastStart MasterMix with ROX (Roche Diagnostics) according to manufacturer’s instructions on Rotor-Gene 3000 (Corbett Research, Sydney, Australia) machine. The primers (final concentration 0.3 µM) used for FXYD5 were: forward 5′-CTCTAGTGACAGATCCAGAG (Tm 53.7 °C), reverse 5′-GTGTTCATCATAGAAGAAGG (Tm 57.7 °C), and for GAPDH: forward 5′-CACCAACTGCTTAGCACCC (Tm 63.3 °C), reverse 5′-GCAGGGATGATGTTCTGGA (Tm 63.8 °C).

### Western blotting

30 µg proteins in sample buffer (8 M urea, 2% SDS, bromophenol blue) were separated on an 8 % or 12% SDS-PAGE gel and transferred onto Immobilon-P membrane (Millipore). Non-specific binding was blocked with Odyssey® blocking buffer (LI-COR, Lincoln, NE, USA). The membrane was incubated with 2 µg/mL polyclonal FXYD5 antibody (Acris Origene, Herford, Germany), 25 ng/mL monoclonal anti-beta catenin antibody (E247, Abcam), 250 ng/mL monoclonal anti-E-Cadherin antibody (BD Biosciences, San Jose, CA, USA), 25 ng/mL monoclonal anti-β-actin (Abcam) overnight at 4 °C and washed 3×5 min with Tris-buffered saline with 0.1% Tween-20 (TBS-T). After incubating the membrane in anti-mouse or anti-rabbit secondary antibody (IRDye 680RD or 800CW, LI-COR, 1:10 000) and washing as above, the fluorescence was recorded on Odyssey infrared scanner (LI-COR).

### Flow cytometry

Cell culture media with 10 µM Marimastat (Sigma-Aldrich), 10 µM MMP8 inhibitor (CAS 236403-25-1, Merch Millipore) or vehicle control (DMSO) were added to the cells for 24 h. Control and MMP8 + HSC-3 cells were detached with Versene buffer (0.48 mM EDTA in PBS) and gentle scraping. 200 000 cells per well were plated in a Nunc™ v-bottom 96-well plate (Thermo Fisher Scientific) in 1% bovine serum albumin (BSA) in PBS. The cells were fixed in 4% paraformaldehyde for 15 min on ice. After washing with 1% BSA/PBS three times, the cells were stained with 1 µg/ml polyclonal FXYD5 antibody (Acris Origene) in 1% BSA /PBS for 45 min on ice. 1× PBS and 1 µg/ml rabbit IgG (Dako, Glostrub, Germany) were used as controls. After washing as above, the cells were stained with allophycocyanin (APC)-conjugated anti-rabbit secondary antibody (Invitrogen, 1:200), washed as above and analysed with Accuri™ C6 Plus (BD Biosciences).

### Cell-cell adhesion spheroid assay

The assay was performed as described^66^. Briefly, 1,000 cells per well in octuplicate were plated on a 96-well ultra-low attachment plate (ULA, Corning, Kennebuck, ME, USA). After 3 days, the formed spheroids were embedded in Myogel^25^-Fibrin gel (0.5 mg/ml each) and imaged daily for 4 days using 10× objective with PowerShot S50 (Canon, Ota City, Japan) attached to an Eclipse TS100 (Nikon, Minato City, Japan) microscope. Spheroid size, invasive area (total minus spheroid size) and total area were measured with Fiji^67^.

### Cell-matrix adhesion assay

96-well plates were coated with Matrigel (1 mg/ml, Corning), Myogel (1 mg/ml^25^,) or rat tail collagen type I (35 µg/ml, Corning) and incubated at 37 °C overnight. Before plating the cells, excess coating material was removed by suction. Control and MMP8 + HSC-3 cells were passaged 1:2 and allowed to attach. After culturing for 24 h in serum-free media with 0.5% lactalbumin, 6000 cells per well were plated on the coated wells. After 2 h, unadhered cells were removed by washing twice with PBS and the adhered cells were fixed with 10% trichloroacetic acid (TCA) for 15 min at RT. The plate was rinsed three times with distilled water and left to dry overnight. The adhered cells were stained for 20 min with 0.1% (w/v) crystal violet solution, rinsed and dried. 10% acetic acid was used to elute the colour and absorbance at 544 nm was measured with Wallac Victor^2^ (PerkinElmer, Waltham, MA, USA).

### Cell migration assay

A total of 10,000 cells per well were plated on a 96-well IncuCyte® ImageLock plate (Essen BioScience, Am Arbor, MI, USA) and incubated at 37 °C overnight. SiRNAs were added as described before and after two days, the cell layers were scratched with WoundMaker™ tool (Essen BioScience). The cell migration was followed in IncuCyte® S3 (Essen BioScience) supplied with the Scratch Wound assay module. When indicated, 30,000 double-silenced cells per well were plated and the scratching was performed the following day.

### Clonogenic assay

A total of 100 cells per well in sextuplicate were plated on a 24-well plate (Corning). After 1 week of culturing, the cells were fixed with 4% paraformaldehyde for 10 min at room temperature. After washing with PBS, the cells were stained with 0.1% (w/v) crystal violet solution for 30 min at room temperature and rinsed with water until colourless. The plate was dried and scanned with a V750 Pro scanner (Epson, Suwa, Japan). Total number of colonies was analysed with Fiji^67^.

### Cell proliferation and viability assays

To measure proliferation and viability, 5,000 or 7,000 cells per well correspondingly were plated into a 96-well plate and cultured for 24 h. Cell Proliferation ELISA, BrdU (colorimetric) kit (Roche Diagnostics) was applied according to manufacturer’s instructions to analyse cell proliferation. Absorbances were read at 450 nm by using Wallac Victor^2^ (PerkinElmer). For viability assay, resazurin sodium salt (Sigma-Aldrich) at final concentration of 3 µg/ml was added to the cells, incubated for 3 h and the fluorescence at 560 nm excitation/590 nm emission was recorded on Wallac Victor^2^ (Perkin Elmer).

### Statistical analysis

All functional cell experiments were repeated a minimum of three times. IBM SPSS statistics version 25 was used for statistical analyses. For all assays, the normality and variance of the data was analysed and either Student’s t-test or Mann-Whitney U-test was applied. Statistical significance was assessed to *p* < 0.05.

## Supplementary information

Supplementary file

Supplementary tables
