# Supplementary material for: MMP8 increases tongue carcinoma cell–cell adhesion and diminishes migration via cleavage of anti-adhesive FXYD5
Source: Oncogenesis. 2021 May 31;10(5):44. doi: 10.1038/s41389-021-00334-x (PMC8167110; doi:10.1038/s41389-021-00334-x)
Supplement: Supplementary file 1 — Supplementary file [file 41389_2021_334_MOESM1_ESM.pdf]

Supplementary file - Juurikka *et al.* "MMP8 increases tongue carcinoma cell-cell adhesion and diminishes migration via cleavage of anti-adhesive FXYD5"

Supplementary figures

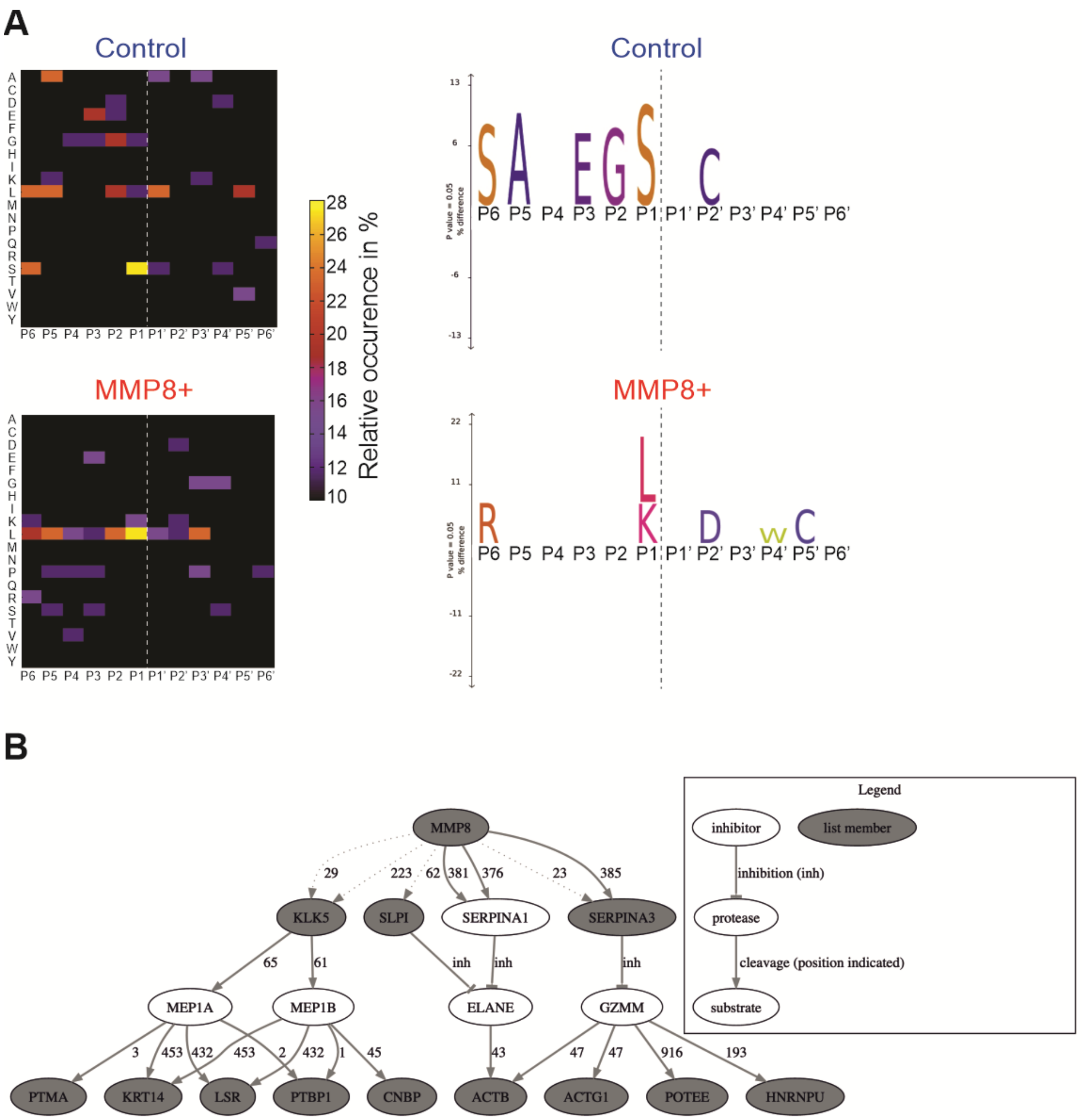

Figure S1. Cleavage sequence specificity profiles of the TAILS data of control and MMP8+ cells. A) Identified cleavage sites are summarized as heat maps showing relative occurrence (left panels) and as iceLogos, showing percent difference compared to natural amino acid abundance (right panels). P6 to P6′ subsite positions are shown on the x axes; plotted amino acids are indicated on the y axes with one-letter codes. In iceLogos, significantly over-represented amino acids are shown above the x axis, under-represented residues below the x axis. B) Protease web of MMP8 generated using PathFINDER.

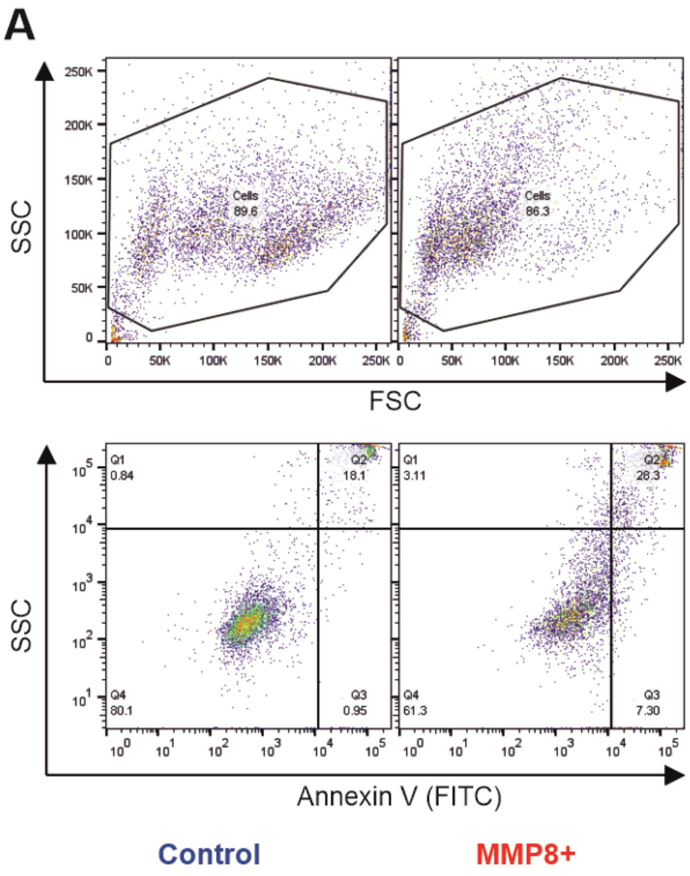

Cell line	Alive (PI-, AnnV-)	Early apoptosis (PI-, AnnV+)	Late apoptosis (PI+, AnnV+)	Total Apoptosis
Control	80.1%	0.95%	18.1%	19.1%
MMP8+	61.3 %	7.3%	28.3%	35.6%

Figure S2. Analysis of apoptosis in MMP8+ and control HSC-3 cells with flow cytometry. Apoptosis assay was performed with eBioscience ™ Annexin V-FITC Apoptosis Detection Kit (Invitrogen) per manufacturer's instructions for control and MMP8+ HSC-3 cells grown to confluency and detached gently with Versene buffer (0.48 mM EDTA in PBS). Analysis was performed with Accuri™ C6 Plus flow cytometer (BD Biosciences). A) Presentative results.

A

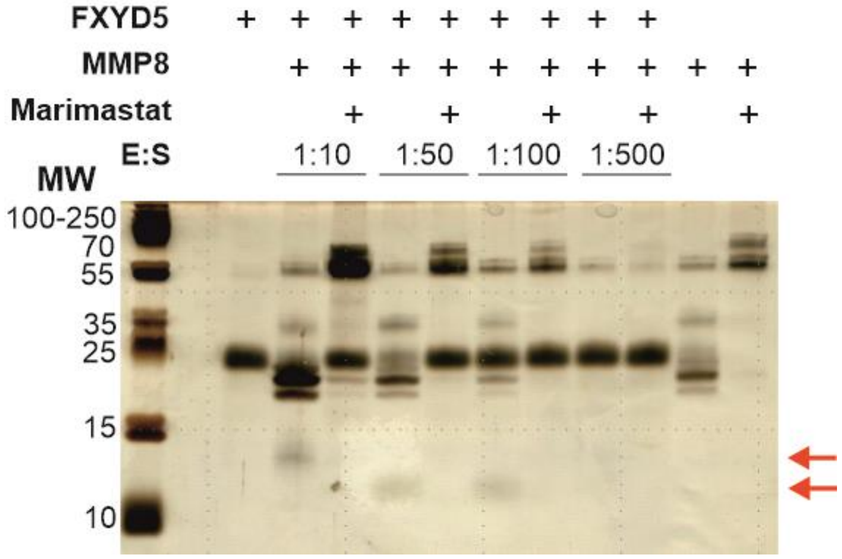

Figure S3. Cleavage assay of recombinant FXYD5 with and without APMA-activated recombinant MMP8 or 10 µM MMP inhibitor Marimastat with enzyme:substrate (E:S) ratios of 1:10-1:500.

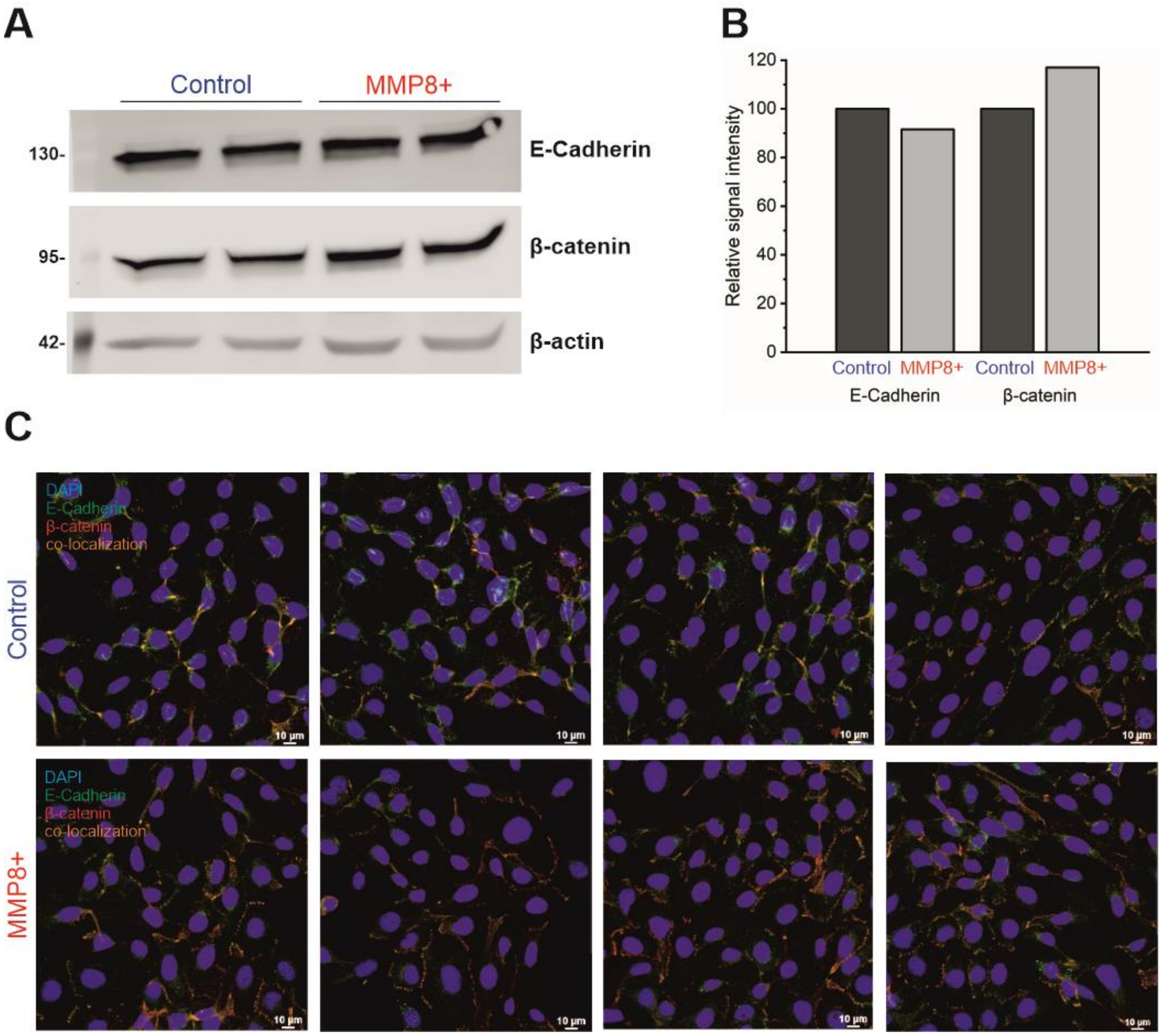

Figure S4. Analysis of E-cadherin and β-catenin expression in control and MMP8+ HSC-3 cells. A) Western blot analysis for E-cadherin and β-catenin in control and MMP8+ HSC-3 cells. B) Quantification of band intensity in western blot relative to β-actin intensity. C) Localisation of E-cadherin and β-catenin in control and MMP8+ cells as analysed by immunofluorescence. 10,000 cells were plated on poly-L-lysine (0.1 mg/ml, Sigma-Aldrich) coated glass chamber slides and the next day the cells were fixed with 4% paraformaldehyde and permeabilised with 0.1% Triton-X. Nonspecific binding was blocked with 10% goat serum after which the slides were incubated with rabbit monoclonal recombinant anti-beta catenin antibody [E247] (1:250, Abcam) and AlexaFluor546-conjugated goat anti-rabbit antibody (1:1000, Invitrogen) was applied as secondary antibody. For double staining, the blocking, primary and secondary antibody incubations were repeated with mouse monoclonal anti-E-Cadherin antibody (1:100, BD Biosciences) and AlexaFluor488-conjugated goat anti-mouse secondary antibody (1:1000, Invitrogen). Cell nuclei were stained with 4′,6-diamidino-2-phenylindole (DAPI). Doublestaining was visualised with C2+ confocal microscope (Nikon) in 60x magnification. Scale bar 10 µm.

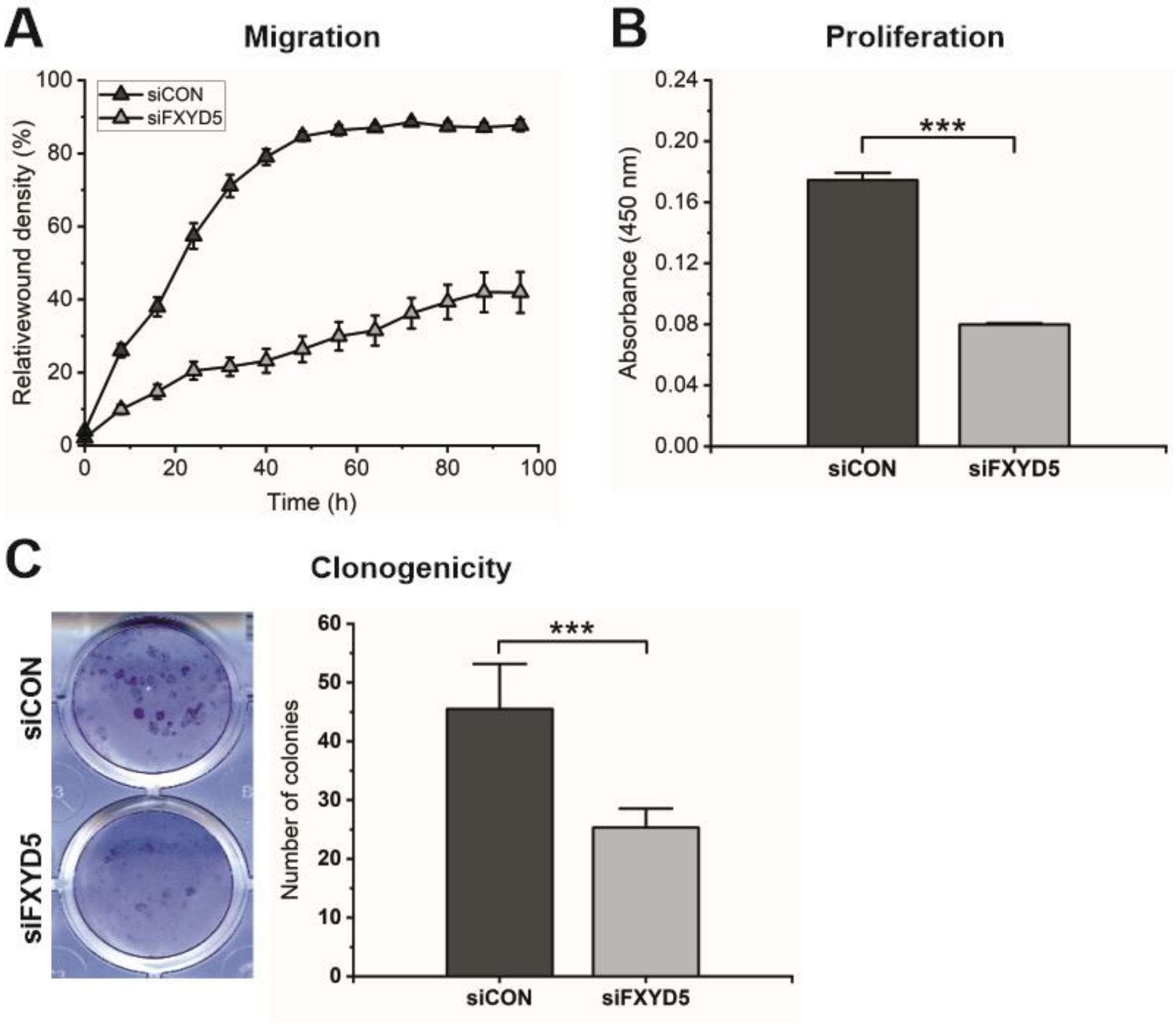

Figure S5. Functional assays for SCC-25 cell line after FXYD5 silencing. A) Proliferation analysed with BrDu. *** p≤0.001. B) Migration analysed with IncuCyte Scratch wound assay. C) Clonogenicity analysed with clonogenic assay including representative images. *** p≤0.001.

Supplementary methods

Immunohistochemistry

Formalin-fixed paraffin-embedded (FFPE) tumour tissue samples from oral tongue squamous cell carcinoma (OTSCC) patients treated in the Oulu University Hospital between 2009-2016 were obtained (n=44). The use of the OTSCC patient samples and the data inquiry were approved by the Ethics Committee of the Oulu University Hospital (#49/2010) and by the National Supervisory Authority for Welfare and Health, Valvira (6865/05.01.00.06/2010). 4 µm sections were cut from the FFPE tissues, deparaffinized and rehydrated (Tissue-Tek®DRS™2000). Tris-EDTA buffer (pH 9) was used for antigen retrieval with microwaving for 15 minutes on Milestone T/T Mega Multifunctional Microwave Histoprocessor (Shelton). After cooling, the slides were washed with PBS–Tween® 20 (PBS-T). 10 min incubation at room temperature with Dako REAL™ peroxidase blocking solution (Dako) was used for blocking endogenous peroxidase activity. After washing as described before, sections were incubated for 30 min at room temperature in Dysadherin (FXYD5) (center) rabbit polyclonal antibody (OriGene) diluted 1:100 in Dako REAL™ Diluent (Dako). After another washing, the sections were incubated horseradish peroxidase (HRP) conjugated secondary antibody (Dako REAL™ Envision™) for 30 min at room temperature. DAB was used as chromogen and counterstaining was performed with Mayer's haematoxylin.
